# Ultrasonograph and Clinical Quantitative Characterization of Tendinopathy by Modified Splitting in a Goat Model

**DOI:** 10.1100/2012/472023

**Published:** 2012-09-10

**Authors:** A. Kavaguchi De Grandis, C. Boulocher, E. Viguier, T. Roger, S. Sawaya

**Affiliations:** Université de Lyon et VetAgro Sup, Campus Vétérinaire de Lyon, UPSP ICE, 69280 Marcy l'Etoile, France

## Abstract

A tendinopathy is a clinical condition characterized by activity-related pain, focal tendons tenderness, and intratendinous imaging changes. This study characterizes a surgically induced tendinopathy in a goat model with a noninvasive *in vivo* longitudinal followup based on physical examination and US. Cross-sectional area (CSA) is the most objective feature for the evaluation of tendinopathy in correlation with clinical findings. The deep digital flexor tendon (DDFT) of the left hind limb of six goats was isolated and scarified by a modified splitting. Pain and lameness at walk and trot were evaluated. External width and thickness of tendon region were measured by calipers. CSA and the ratio lesion/tendon CSA were obtained at days 0, 7, 21, 42, and 84 by US. The highest value of global functional score was obtained at day 7, then decreased until day 40 and was not significantly different from day 0 at the end of the study. The external width recovered a normal value at the end of the study, but the external thickness was still significantly increased (*P* < 0.05). Peritendinous oedema was observed at day 7, but intratendinous lesions were visible only at day 21 as a focal hypo to anechoic area. At day 84, two tendons still presented visible lesions. US examination was reproducible, specific, and provided complementary information to the global functional score. A standardized focal tendinopathy was induced in goats. This experimental model of focal tendinopathy could be used to study the effect of different treatments.

## 1. Background 

A tendinopathy is a clinical condition characterised by activity-related pain, focal tendons tenderness, and intratendinous imaging changes requiring a recovery time of at least three to six months with first-line conservative management [1, 2].

In human and veterinary medicine, the musculoskeletal system is of special interest, as its damages are often fatal for adequate sportive performance. The incidence of tendinitis in sport horses has been reported to be between 11% and 46%, and recurrent injuries were found in 43 to 90% of the cases [3–5].

Injuries to the distal aspect of the limbs are common in horses and frequently involve injury of the flexor and/or extensor tendons [4, 6]. 

There is no consensus on a “gold standard” among the experimental models of tendinopathy: model requirements change as knowledge of the disease increases. Nonhuman primates, horses, goats, dogs, rabbits, rats, and mice have previously been used [1, 7]. Large animal models present the advantage to having naturally occurring tendonitis [1]. In addition, clinical examination and lameness assessment are easier to perform on large rather than on small animals. 

Tendinopathies can be induced chemically by intratendinous injection of collagenase, PGE1, PGE2, corticosteroid, or cytokines [1, 7–9]. Recently new models have been developed *ex vivo*: cyclical loading, creep loading, and stress deprivation, and *in vivo*: electrical muscle stimulation, downhill and uphill treadmill running, fatigue loading, and disuse [7]. A surgical model (tenectomy) has been described in goats to study acute tendon rupture and healing [10]. 

In human and veterinary medicine, the usefulness of ultrasonography (US) in the diagnosis and in the characterisation of soft tissues injuries of the locomotor system has been widely demonstrated [11–15]. It is considered as an essential imaging modality to evaluate the healing process of tendon lesions [15–17], and its real-time dynamic capability offers a major advantage compared with other imaging techniques [11]. US allows early detection of tendons lesions and the evaluation of their surrounding, in particular, synovial sheath lesions [18]. Analyses of US images use both quantitative and qualitative measurements to assess injuries, including echogenicity, fibre alignment, size, shape, margination, and tendon position. Cross-sectional area (CSA) is the most objective feature for the evaluation tendon pathology and can be assessed accurately with US [19–21]. 

US is also used to adjust exercises programmes during rehabilitation, with adaptation of the treatment for specific injuries [14] and to determine the readiness return to exercise and competition [12]. According to Reef 1998 [22], a CSA increase of more than 10% is suggestive of reinjury during the rehabilitation period. Nevertheless, the diagnostic values of imaging findings have to be correlated with the clinical findings [23].

The present study is part of a larger investigation on an experimental tendinopathy in a goat model. The objectives were to use US to establish the normal CSA of the metatarsal region of the goat model and to perform a noninvasive longitudinal followup of the tendon injury and healing (physical examination and US). 

## 2. Methods

The study was approved by the Ethical Committee on Animal Experiments of VetAgro Sup, Lyon, France (No. 1020/2010).

### 2.1. Animal Experimental Model

Six female adult goats weighing around 55 Kg and aged between 2 and 6 years old were used in the study. The goats were group-housed in a paddock at the Institut Claude-Bourgelat, Marcy l'Etoile, France, with hay and water ad libitum. Preentrance sanitary exams were performed to make certain goats not affected by any pathology which could interfere with the study. The acclimation period was of 21 days. Then, to induce a surgical focal tendinopathy, a modified splitting was performed on the left hindlimb deep digital flexor tendon (DDFT) in the metatarsal region. 

### 2.2. Surgical Procedure

Goats were premedicated by subcutaneous injection of buprenorphine (0.02 mg/Kg). Anaesthesia was induced by intramuscular injection of a combination of xylazine (Rompun 2%, Bayer, 0.05 mg/kg) and ketamine (Imalgene 1000, Merial, 2.5 mg/kg) and maintained with isoflurane 2% in oxygen after intubation. Goats were given prophylactic antibiotic therapy prior to surgery by intramuscular injection of amoxicillin (15 mg/kg). 

The left hind limb was operated on under sterile conditions. A three centimetre incision was made on the skin at the middle of the metatarsal region on the plantaro-lateral aspect. Then the DDFT was isolated and scarified as follows: 1/longitudinal splitting (1 cm) of the tendon and of the tendon sheath, 2/360 degrees rotation of the scalpel which created a “window” within the tendon fibres. All animals were operated on by the same person, using exactly the same technique.

**Figure 1:**
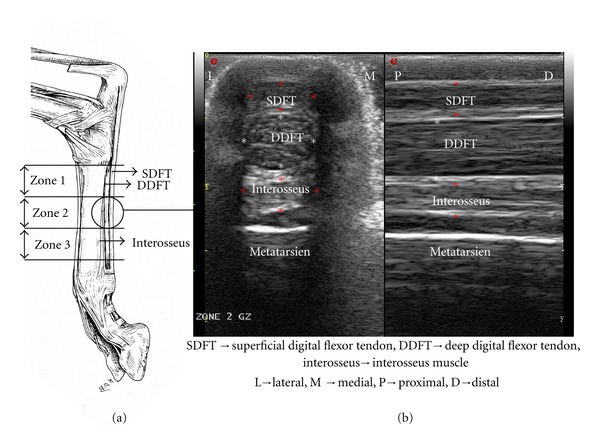
(a), anatomy of the goat's hind limb. (b), transversal and longitudinal US images of zone 2.

The surgical wound was closed by separated points with Ethylon 2-0 and cleaned with vetedine solution (vetoquinol). The operated limb was then immobilized with a thick, well-packed compression bandage (Robert Jones type) for four days. The nonoperated limb was not immobilized. Goats were allowed free activity in their paddock and veterinarians closely monitored their recovery. Postoperative analgesia was managed by a 0.02 mg/Kg subcutaneous injection of buprenorphine twice a day for two days. See Figure 1.

### 2.3. Clinical Followup

#### 2.3.1. Pain Assessment

Pain threshold was defined as the minimum pressure applied through the 1 cm² tip that induced pain, as described by Rompe et al. [24, 25], at the level of zone 2 with an algometer (Dolormeter, EMS, Nyons Suisse). The essence of pressure algometry is that increasing pressure applied to the part of the body to be investigated, and the outcome is the patient's reaction to the pressure. The algometer was calibrated to give an integer between 0 and 5 (5: no pain, 0: maximal pain). 

Lameness at walk and trot was evaluated semi-quantitatively according to Millis [26], Table 1.

A global functional score was calculated based on lameness at walk and trot and normalized (score of lameness at walk + score of lameness at trot)/2.

External width (distance between the skin and the plantar edge of the metatarsus) and thickness (distance skin to skin from medial to lateral) of the region of tendons were measured at the level of the surgery to follow up oedema and inflammation. The same person performed all the measurements with a digital calliper (“Casto” 0–150 mm, Castorama, 59175 Templemars, France).

### 2.4. *In Vivo* Ultrasonography (US)

#### 2.4.1. US Protocol

Ultrasonography of the operated limb was performed *in vivo* without sedation prior to the surgery at days 0 and then 7, 21, 42, and 84 days after the surgery. A protocol for US of the goat metatarsal region was created and standardised, based on the routine US examination protocol used for horse metatarsal region at VetAgro Sup. US examinations were performed with a MyLab70 X-Vision (Esaote) with a linear probe (6 to 18 MHz—Diasus Dynamic Imaging Ltd). Left metatarsal region was clipped; isopropyl alcohol and ultrasound coupling gel were applied to ensure good contact between the skin and the transducer. Examinations were performed with the goats weight-bearing on all four limbs. 

Each metatarsal region was divided into three main zones of equal size from the tarsometatarsal joint to the bifurcation of the DDFT, that is, above the ergots. Zone 1 covered the proximal third of the metatarsal region; Zone 3 covered the distal third; Zone 2 was in between and corresponded to the splitting and to the examined zone. US exam was performed before surgery for each animal to record a control image. 

#### 2.4.2. US Measurement of the DDFT Lesion

In Zone 2, quantitative measurements of the cross-sectional areas (CSA) of DDFT were done. The measurement was performed when the image showed the smallest CSA (i.e., when the ultrasound probe was perpendicular to the tendon thus preventing from the anisotropic artefact). The lesion areas were measured and the ratio lesion/tendon cross-sectional areas were recorded for each tendon. Measurements and ratio were calculated directly on the US machine's software.

Examinations were carried out by the same operator to avoid interobserver error. The lesion was tagged on the images to help for the next examination and to assure repeatability.

### 2.5. Statistics

The data were statistically analysed with Excel (Microsoft Office) and R 2.11 for Windows. The effect of time was assessed by comparison of values between the different time points using paired *t*-test when distribution was normal and Wilcoxon test when it was abnormal. 

## 3. Results

All animals were operated on under the same conditions. Their waking was calm and uneventful. No wounds complications were observed after surgery. All the goats completed the study. The operated area of tendon could be identified and followed up by the same methods for all animals. 

### 3.1. Clinical Examination

**Figure 2:**
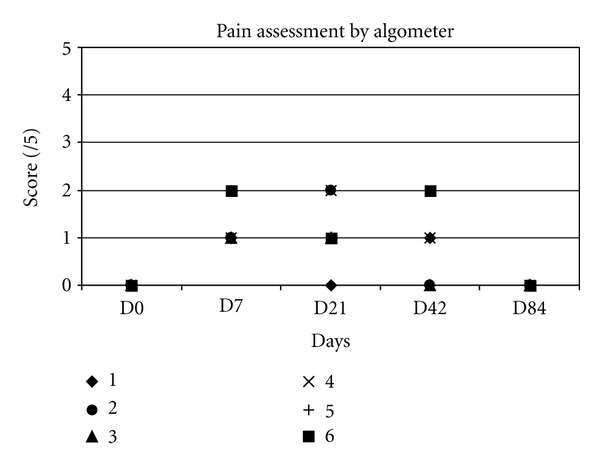
Pain assessment by algometer.

The results of local pain assessment are displayed in (Figure 2).

No animal showed local pain before surgery. At day 7, the highest value of pain was obtained with a significant difference compared to day 0 (*P* < 0.05). Between days 7 and 21, the scores stayed high and then decreased progressively until the end of the study when they returned to zero.

**Figure 3:**
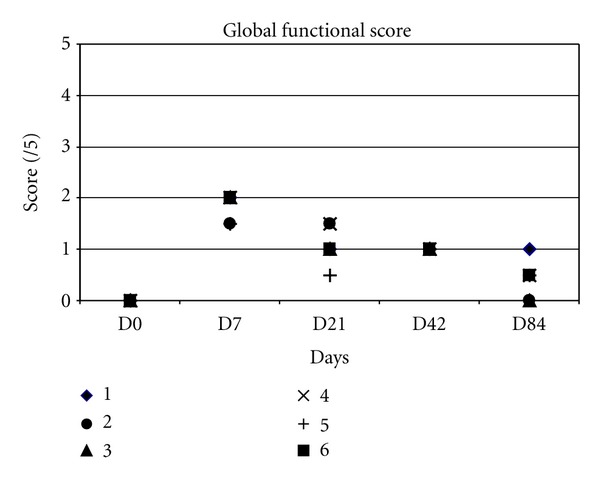
Global functional score of lameness.

The global functional scores are displayed in (Figure 3).

All goats presented the highest score of lameness at day 7 with a significant difference compared to day 0 (*P* < 0.05). Between day 7 and 84, they presented a diminution of their scores. And at day 84, there was no difference compared to day 0.

**Figure 4:**
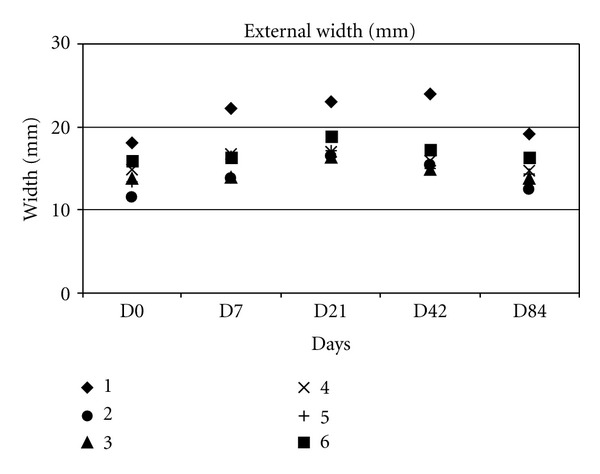
Change in external width.

**Figure 5:**
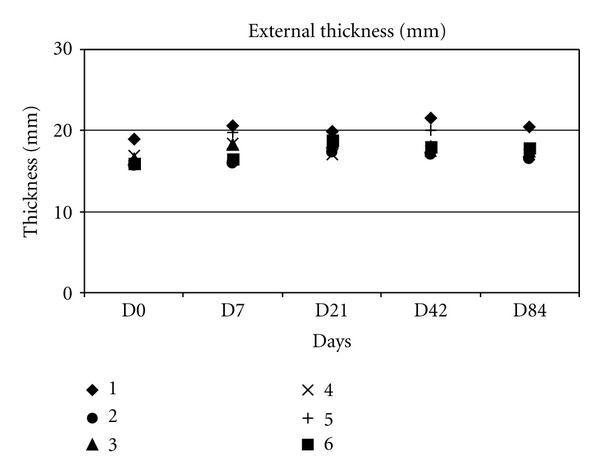
Change in external thickness.

Measurements of external width and thickness are represented in (Figures 4 and 5).

At day 0, the external width of zone 2 was 14.59 ± 2.26 mm, increased until day 21 (18.13 ± 2.58 mm), and then decreased until the end of the study (17.09 ± 3.46 mm at day 42 and 15.11 ± 2.33 mm at day 84). The difference was significant between day 7 and day 0 (*P* = 0.03) but not significant between days 7 and 42 (*P* > 0.3 between days 42 and 7 and *P* > 0.06 between days 42 and 21). At day 84, the external width was not significantly different from its initial size.

At day 0, the thickness of zone 2 was 16.71 ± 1.19 mm, increased until day 42 (18.69 ± 1.74 mm) and decreased until day 84. At day 84, the size of zone 2 was not significantly different from its initial size.

### 3.2. *In Vivo* Ultrasonography (US)

All tendons presented similar images at day 0 (Figure 8). At day 7, on the longitudinal view, the operated tendons appeared to be increased in volume, and a discontinuity of the parallel aspect of the margins was noticed. On this view, we could not detect/visualize the lesions. On the transverse view, a convex deformation of the tendon was apparent around the operated area. At day 7 and at day 21, focal lesions were observed and quantified.

**Figure 6:**
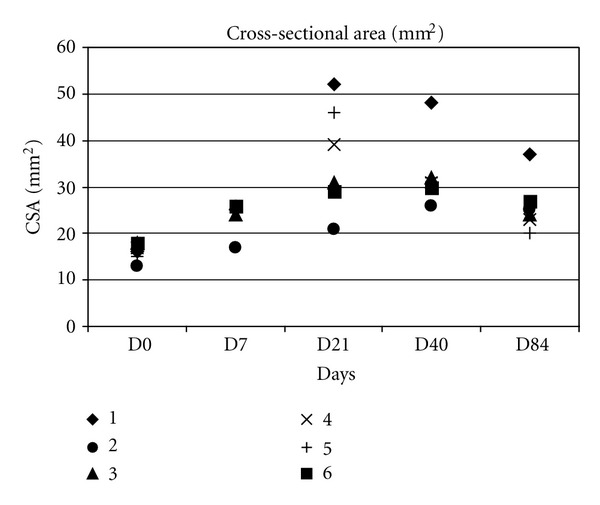
Change in CSA.

**Figure 7:**
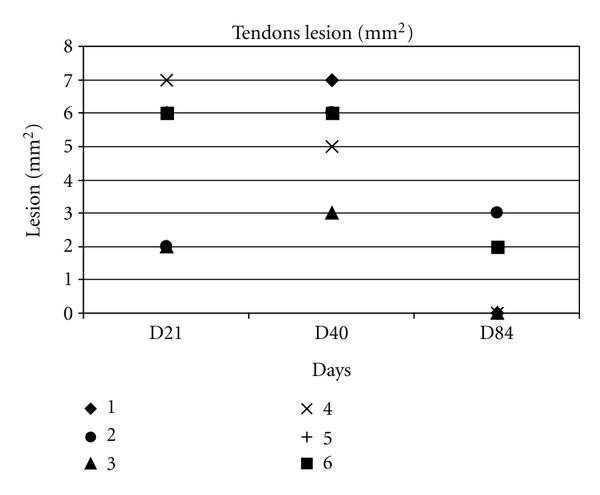
Change in measurements of tendon's lesion.

Measurements of CSA and lesion areas are presented in (Figures 6 and 7).

**Figure 8:**
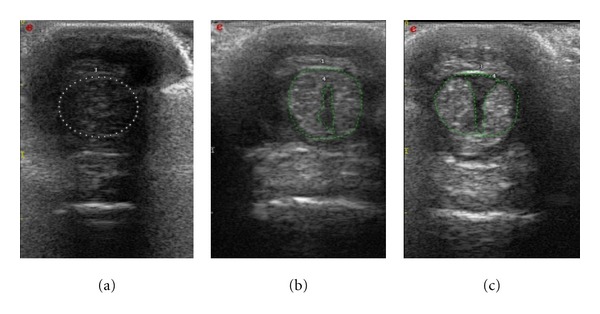
Transversal image at day 7 (a), day 21 (b), and day 42 (c), when the lesion is bigger than at day 21.

At day 7, all tendons presented an increased CSA (24 ± 3 mm²) compared to day 0 (16 ± 2 mm²) with a significant difference (*P* < 0.001). Peritendinous oedemas (anechoic image around the tendon) were observed in all the operated hindlimbs without visible intratendinous lesions (Figure 8). At day 21, the CSA (36 ± 12 mm²) was at its largest size with a significant difference compared to the day 0 (*P* < 0.05). Lesions were identified and measured in all tendons at day 21 (4.8 ± 2.2 mm² or 13.2 ± 5.3% of CSA) (Figure 8). At day 42, CSA was still increased (33 ± 8 mm²) compared to day 0 (*P* < 0.005) and day 7 (*P* < 0.05) but was lower than at day 21 (*P* > 0.5) (Figure 8). 

Lesions appeared hypo to anechoic at day 42 with a larger area than at day 21 (5.5 ± 1.4 mm² or 17 ± 4.8%). At day 84 two of six tendons still presented an increased CSA (26 ± 6 mm²) compared to day 0 (*P* > 0.01). 

The highest values of the operated zone external width, thickness and CSA were at day 21. The highest value of the global functional score was at day 7. 

No correlation was observed between the global functional score and the external size of the operated zone or its US area.

## 4. Discussion 

This study presents a standardized experimental model of surgically induced tendinopathy: lesions were performed by the same operator, the same technique, and the same material. All the operated tendons presented the same gross changes, and lesions were seen ultrasonographically in all animals at day 21 demonstrating the homogeneity of the lesions. 

As in other species, US is a relevant, reliable, and inexpensive examination. It allowed *in vivo*, noninvasive, and longitudinal monitoring of the lesions of tendons and to work in sedation-free conditions, as described by other authors [17, 27]. However, we could not use the longitudinal images to quantify the lesion. We supposed that the elasticity of the tendon did not allow us to observe the lesion itself, but we could observe its consequences.

In human and veterinary medicine, it is described that almost all tendons have an identical echostructure. Here, we noticed the longitudinal and axial US aspects of the goat tendon were similar to the description of Sans et al. [18]. In longitudinal view, they present a broadly hyperechoic, fibrillar structure parallel to the major axis of the tendon, corresponding to bundles of collagen fibres that are surrounded by a structure of middle echogenicity. In axial/transverse view, the tendon is an oval structure, limited by a hyperechoic border, and presenting paint strokes of regular echoes, with a synovial sheath which appears like a hypoechoic fine band [18]. In goats, the DDFT appears less homogeneous than horses DDFT making the US assessment of the fibre's alignment difficult.

Another difficulty of tendon US imaging is the artefact of anisotropy that occurs when the incident ultrasound wave is not perpendicular to the axis of the tendon or parallel to him in the longitudinal plane, so an aspect hypoechoic of the tendons is observed [12, 18]. To prevent anisotropic artefact, we performed measurement of CSA on the image where it appeared the smallest and with the highest signal intensity (i.e., US probe perpendicular to the tendon long axis).

In horses, the literature shows a slight tendency for tall horses to have a higher tendon width and thickness [19, 23]. No correlation was observed between the weight and size of the animals and their tendon's CSA or external size. Variations in the CSA of tendons and ligaments of clinically normal horses have been described in a number of breeds [21, 28–30]. The variation of the CSA observed could be expressed by the individual variation of CSA of the healthy tendon. No published references for the echogenicity or the CSA of goat's tendons were found. Interestingly, we observed that goat's DDFT was more heterogeneous than in horses making the qualitative evaluation of its echogenicity difficult and not reliable. While peritendinous oedemas (anechoic image around the tendon) were observed in all operated legs, no intratendinous lesions could be recognized at day 7 by US. An increase in the tendon mass and CSA appears to occur during the modelling stage of a tendinopathy [31]. This was also found at day 84. In horses, after flexor tendons injuries, at least 6 weeks are required for the tendon to gain sufficient strength to bear weight [6]. Indeed, different authors mention a recovery time of three to six months [1, 2]. However, tendon US morphology does not necessarily correlate with clinical symptoms [32]. 

The goat is relevant as an experimental model in the study of tendinopathy as they are easy to house and to handle and are cheaper than horse or dog models. In addition, their size allows for performing similar examination to be performed on humans, horses, or dogs (clinical and imaging exams). 

Clinical examination and ultrasonography were easy to perform *in vivo *with gentle handling only and provided complementary information on the goat's tendon healing process. The normal US images and tendon CSA presented here could be used as a reference for following studies such as the evaluation of different therapies. 

## Figures and Tables

**Table 1:** A global functional score was calculated based on lameness at walk and trot and normalized (score of lameness at walk + score of lameness at trot)/2.

Score of lameness—walk and trot [26]	
0	Normal (without lameness)
1	Discrete lameness (lameness barely visible and intermittent)
2	Obvious lameness (visible lameness)
3	Severe lameness (evident lameness)
4	Lameness with intermittent support removal
5	Constant support removal
